# Dissociation between Brain Amyloid Deposition and Metabolism in Early Mild Cognitive Impairment

**DOI:** 10.1371/journal.pone.0047905

**Published:** 2012-10-24

**Authors:** Liyong Wu, Jared Rowley, Sara Mohades, Antoine Leuzy, Marina Tedeschi Dauar, Monica Shin, Vladimir Fonov, Jianping Jia, Serge Gauthier, Pedro Rosa-Neto, the Alzheimer’s Disease Neuroimaging Initiative

**Affiliations:** 1 Translational Neuroimaging Laboratory, Douglas Hospital, McGill University, Montreal, Quebec, Canada; 2 McGill Centre for Studies in Aging, McGill University, Montreal, Quebec, Canada; 3 Department of Neurology, Xuan Wu Hospital, Capital Medical University, Beijing, China; 4 McConnell Brain Imaging Centre, Montreal Neurological Institute, McGill University, Montreal, Quebec, Canada; Nathan Kline Institute and New York University School of Medicine, United States of America

## Abstract

**Background:**

The hypothetical model of dynamic biomarkers for Alzheimer’s disease (AD) describes high amyloid deposition and hypometabolism at the mild cognitive impairment (MCI) stage. However, it remains unknown whether brain amyloidosis and hypometabolism follow the same trajectories in MCI individuals. We used the concept of early MCI (EMCI) and late MCI (LMCI) as defined by the Alzheimer’s disease Neuroimaging Initiative (ADNI)-Go in order to compare the biomarker profile between EMCI and LMCI.

**Objectives:**

To examine the global and voxel-based neocortical amyloid burden and metabolism among individuals who are cognitively normal (CN), as well as those with EMCI, LMCI and mild AD.

**Methods:**

In the present study, 354 participants, including CN (n = 109), EMCI (n = 157), LMCI (n = 39) and AD (n = 49), were enrolled between September 2009 and November 2011 through ADNI-GO and ADNI-2. Brain amyloid load and metabolism were estimated using [^18^F]AV45 and [^18^F]fluorodeoxyglucose ([^18^F]FDG) PET, respectively. Uptake ratio images of [^18^F]AV45 and [^18^F]FDG were calculated by dividing the summed PET image by the median counts of the grey matter of the cerebellum and pons, respectively. Group differences of global [^18^F]AV45 and [^18^F]FDG were analyzed using ANOVA, while the voxel-based group differences were estimated using statistic parametric mapping (SPM).

**Results:**

EMCI patients showed higher global [^18^F]AV45 retention compared to CN and lower uptake compared to LMCI. SPM detected higher [^18^F]AV45 uptake in EMCI compared to CN in the precuneus, posterior cingulate, medial and dorsal lateral prefrontal cortices, bilaterally. EMCI showed lower [^18^F]AV45 retention than LMCI in the superior temporal, inferior parietal, as well as dorsal lateral prefrontal cortices, bilaterally. Regarding to the global [^18^F]FDG, EMCI patients showed no significant difference from CN and a higher uptake ratio compared to LMCI. At the voxel level, EMCI showed higher metabolism in precuneus, hippocampus, entorhinal and inferior parietal cortices, as compared to LMCI.

**Conclusions:**

The present results indicate that brain metabolism remains normal despite the presence of significant amyloid accumulation in EMCI. These results suggest a role for anti-amyloid interventions in EMCI aiming to delay or halt the deposition of amyloid and related metabolism impairment.

## Introduction

Alzheimer’s disease (AD) is characterized by a progressive accumulation of amyloid plaques, neurofibrillary tangles and neuronal depletion associated with a slow deterioration of cognition and functional status [1]. Numerous technological advances have made possible the quantification of amyloid accumulation and neurodegeneration in vivo using imaging and fluid biomarkers. In AD, biomarkers are classified as biomarkers of amyloid accumulation (i.e CSF Aβ_1–42_, [^11^C] Pittsburgh compound B (PIB) Positron Emission Tomography (PET), [^18^F]AV45 PET) and neurodegeneration (i.e CSF tau, [^18^F] fluorodeoxyglucose(FDG) PET and structural MRI) [2], [3].

**Figure 1:**
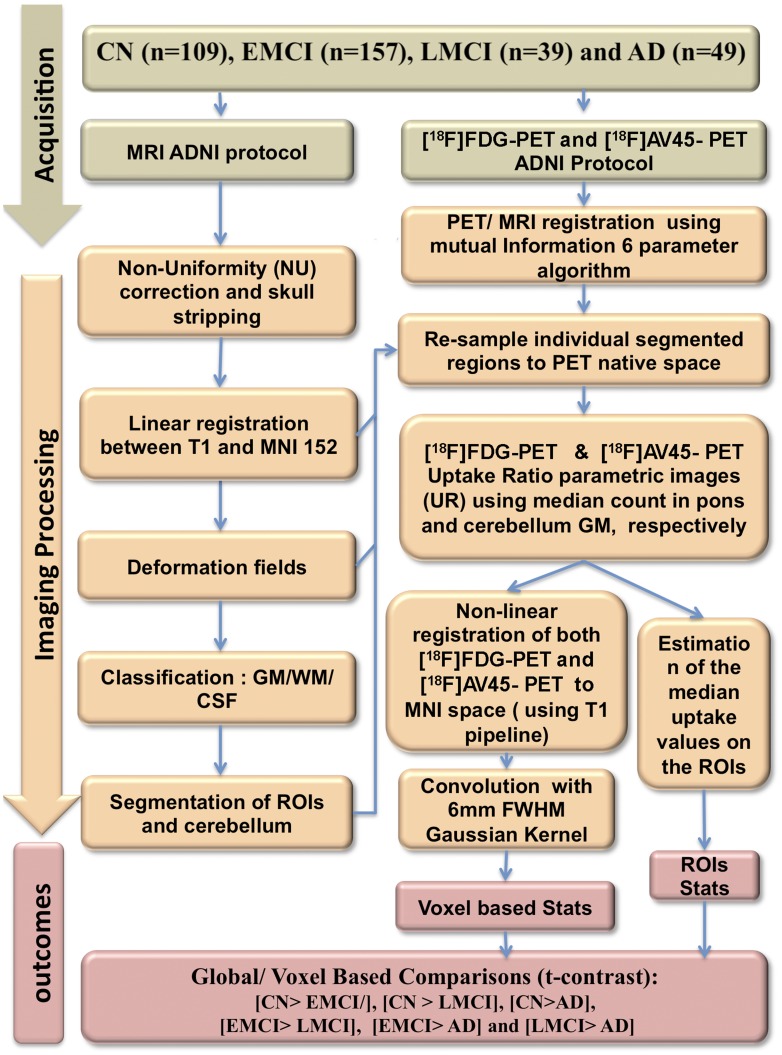
Summary of the imaging analysis methods.

Jack [2] proposed a dynamic biomarker model of Alzheimer’s based on vivo biomarker observations conducted in elderly normal individuals as well as in mild cognitive impairment (MCI) and AD dementia patients. Jacks’ model corroborates the amyloid cascade hypothesis, which posits that the accumulation of β-amyloid acts as an initiating ‘upstream’ event leading to ‘downstream’ events such neurodegeneration and subsequent cognitive impairment [2], [4]. The dynamic biomarker model of AD describes amyloid accumulation as the dominant biomarker in individuals with predementia, while the combination of amyloid accumulation and neurodegeneration characterize the dementia stage of AD [2]. Moreover, this model predicts a plateau of amyloid accumulation and the onset of brain neurodegeneration as part of the MCI stage of AD [2]. However, the dynamics of amyloid accumulation and neurodegeneration within the MCI stage are not well understood.

Based on the data analysis conducted by the National Alzheimer’s Coordinating Center (NACC), the concept of early MCI (EMCI) and late MCI (LMCI) was first introduced by the Alzheimer’s Disease Neuroimaging Initiative (ADNI)-Go and ADNI-2 the distinction being made on the basis of modest or advanced impairment of delayed recall of logical memory [5], [6]. The concept of EMCI will bridge the gap between normal elderly and LMCI subjects who are more amnestic than EMCI subjects [5]. It should be emphasized that while EMCI subjects still meet criteria for amnestic MCI, they represent a very early point in the clinical spectrum of AD [5], [6]. The primary purpose of this classification in ADNI-Go and ADNI-2 is to elucidate the disease mechanisms present at a very early stage of AD (i.e. EMCI), with the attendant objective of improving the efficiency of disease-modification interventions [5], [6].

Although several studies have focused on MCI and healthy elderly control, the status of brain amyloid deposition remains unknown at the early stage of MCI. The study from Australian Imaging Biomarkers and Lifestyle Research Group showed that the amyloid load of subjects with subjective cognitive impairment (SCI), a syndrome characterized by subjective memory complaint but no objective memory impairment, was similar to healthy elderly controls, and thus lower than levels found in MCI and AD [7]. Longitudinal studies using [^11^C]PIB PET have shown no uptake differences during the follow-up of MCI patients who eventually converted to AD, suggesting that amyloid load plateau has been already reached in the late stage of MCI [8], [9]. In fact, these findings suggest that LMCI may represent an intermediate state between cognitively normal individuals and MCI.

**Table 1:** Demographics and neuropsychological data for all groups.

	CN (n = 109)	EMCI (n = 157)	LMCI (n = 39)	AD (n = 49)	*p* value
**Gender (M/F)**	53/56	90/67	24/15	31/18	0.231
**Age (years)**	78.8±5.9	73.1±7.9^a^	76.2±9.2^d^	75.6±7.8	<0.001
**Education (years)**	16.4±2.8	16.0±2.7	16.4±3.2	16.7±2.8	0.304
**MMSE**	29.1±1.2	28.3±1.5^a^	27.1±2.2a c	21.4±4.7a c e	<0.001
**CDR-global**	0±0	0.5±0.1^a^	0.5±0.1^a^	1.0±0.4a c e	<0.001
**CDR-sum of boxes**	0.1±0.3	1.3±0.7^a^	1.9±1.4a c	5.6±2.5a c e	<0.001
**ADAS-cog**	6.2±4.2	7.9±3.4^a^	12.5±5.7a c	21.8±10.3a c e	<0.001
**Immediate recall of logical memory**	14.8±3.6	11.3±3.0^a^	6.5±3.4a c	4.2±3.0a c e	<0.001
**Delayed recall of logical memory**	14.0±3.7	9.3±2.1^a^	3.5±2.8a c	1.5±2.6a c e	<0.001
**Total immediate recall of** **RAVLT (Trial 1 to 5)**	45.0±10.6	39.3±11.0^a^	30.0±9.2a c	21.1±7.9a c e	<0.001
**Delayed recall of RAVLT**	7.1±4.3	6.1±4.2^b^	2.2±2.8a c	0.7±2.3a c e	<0.001
**Recognition of RAVLT**	12.8±2.3	12.2±2.9	9.1±3.8a c	6.5±4.3a c e	<0.001
**Trail making test A**	34.6±14.6	36.4±13.1	44.4±24.0a c	65.9±38.9a c e	<0.001
**Trail making test B**	82.2±37.1	99.8±57.0^a^	140.1±77.8a c	189.6±83.7a c e	<0.001
**Verbal fluency (animal)**	20.8±5.8	18.8±5.0^a^	16.3±5.8a c	11.0±4.4a c e	<0.001
**Boston naming test**	27.8±4.4	27.2±3.5	24.9±6.8a c	23.2±6.5a c	<0.001
**FAQ**	0.3±1.4	2.3±3.4^a^	5.0±5.4a c	15.8±6.5a c e	<0.001

All values are indicated as mean ± standard deviation except gender. p value indicates the value for the main effect of each group, as assessed with analyses of variance(ANOVA) for each variable except for gender, where a contingency chi-square was performed. Statistics for post hoc 2-by-2 group comparisons are provided as significant differences:

a from CN at p<0.01;

b from CN at p<0.05.

c from EMCI at p<0.01;

d from EMCI at p<0.05;

e from LMCI at p<0.01.

MMSE =  mini mental state examination; CDR = clinical dementia rating scale; ADAS  =  Alzheimer’s disease assessment scale; RAVLT =  Rey auditory verbal learning test; FAQ =  functional activities questionnaire.

The existence of synaptic degeneration in EMCI remains to be clarified, particularly due to the presence of mild but objective memory deficits in these individuals. [^18^F]FDG PET provides qualitative and quantitative estimates of the cerebral metabolic rate of glucose consumption, an index of synaptic functioning and density, which has been proven to be highly related with the clinical symptoms and taken as the symptoms-sensitive measurement in MCI [10], [11]. Several studies have shown that MCI patients displayed cerebral hypometabolism bilaterally in the parieto-temporal areas, posterior cingulate cortex (PCC), medial temporal lobe, and even frontal lobe [10], [12]–[14]. It has been suggested that glucose metabolism is a sensitive measure of change in cognition and functional ability in MCI, and has value in predicting future cognitive decline [10], [14]. Furthermore, MCI subjects with abnormal glucose metabolism were more likely to convert to AD than subjects who had normal glucose uptake [10]–[13]. Therefore, [^18^F]FDG PET provides significant information regarding the clinical characterization of synaptic dysfunction already present in EMCI [3], [15].

The main objectives of the present study are thus to examine global and voxel-based neocortical amyloid burden and metabolism within separate groups of cognitively normal (CN), EMCI, LMCI, and AD. We intend to capture the dynamics of amyloid accumulation and neurodegeneration within the MCI stage. Assuming the premises of the amyloid cascade hypothesis, we expect a predominance of amyloid accumulation in the EMCI phase.

## Materials and Methods

### Database Description

Participants were recruited between September 2009 and November 2011 through ADNI-GO and ADNI-2 from 56 centers in the USA and Canada [16]. The ethics committee at each participating site approved the study protocol. Written consent was obtained from all subjects participating in the study. Inclusion/exclusion criteria for ADNI studies are described elsewhere (https://ida.loni.ucla.edu/login.jsp?project=ADNI).

**Figure 2:**
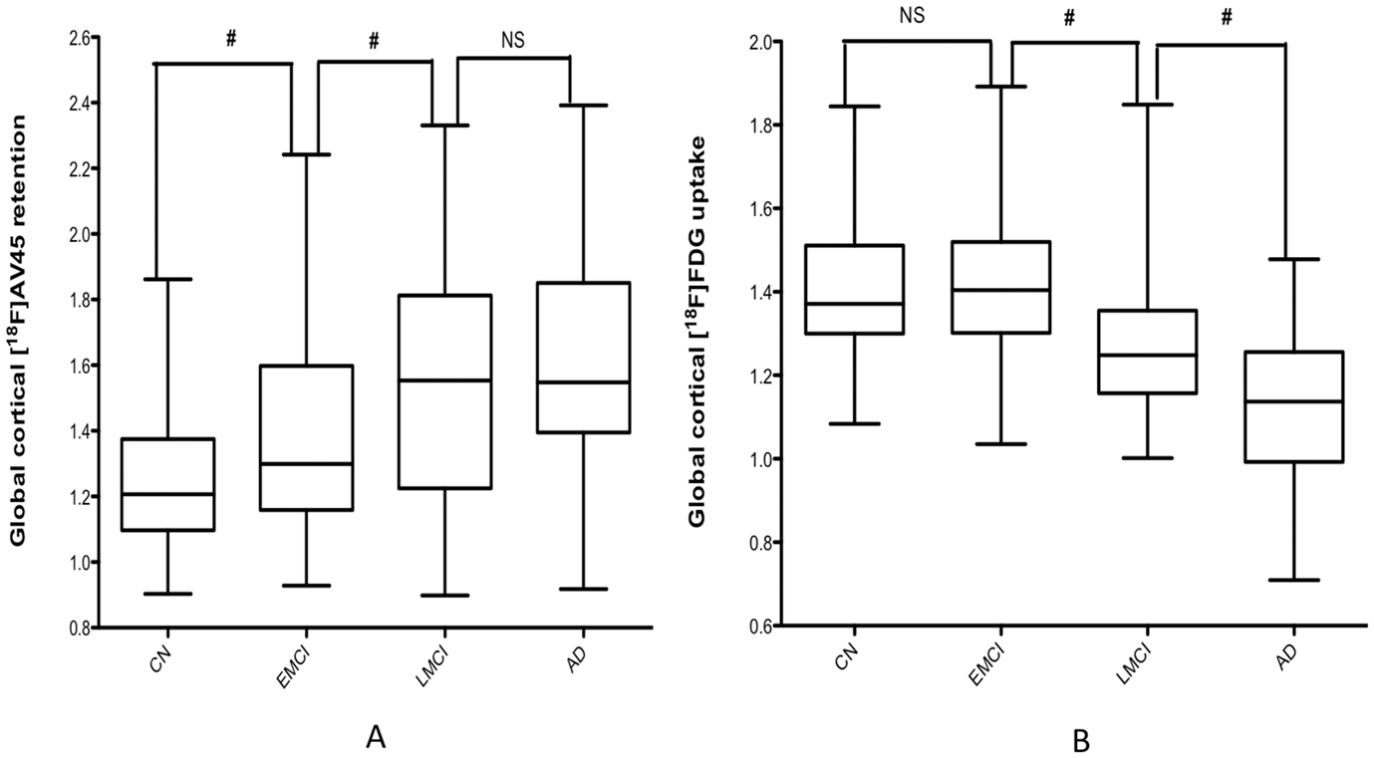
[^18^F]AV45 (left) and [^18^F]FDG (right) average images obtained from controls (CN), early MCI (EMCI), late MCI (LMCI) and Alzheimer’s dementia patients (AD). In [^18^F]AV45, there is a reduction of gray and white matter contrast in EMCI, LMCI and AD in comparison with CN. Note the PCC and IPC [^18^F]FDG SUVR reduction in LMCI and AD in comparison with CN.

**Table 2:** Global [^18^F]AV45 and [^18^F]FDG uptake in all groups.

	NC (n = 109)	EMCI (n = 157)	LMCI (n = 39)	AD (n = 49)	*p* value
**Global [^18^F]AV45 retention**	1.28±0.25	1.40±0.33^a^	1.56±0.36a c	1.62±0.38a c	<0.001
**Global [^18^F]FDG uptake**	1.40±0.15	1.41±0.16	1.28±0.19a c	1.14±0.17a c e	<0.001
**Regional [^18^F]FDG uptake**					
**Left inferior parietal cortex**	1.40±0.16	1.42±0.18	1.28±0.21a c	1.12±0.18a c e	<0.001
**Right inferior parietal cortex**	1.45±0.17	1.46±0.19	1.33±0.23a c	1.16±0.22a c e	<0.001
**Left temporal lobe**	1.35±0.23	1.33±0.16	1.21±0.18a c	1.08±0.17a c e	<0.001
**Right temporal lobe**	1.33±0.15	1.38±0.17	1.28±0.18^c^	1.14±0.18a c e	<0.001
**Left hippocampus**	1.18±0.25	1.06±0.12^a^	0.96±0.15a c	0.88±0.14a c	<0.001
**Right hippocampus**	1.01±0.11	1.02±0.12	0.92±0.14a c	0.84±0.18a c	<0.001
**Left posterior cingulate cortex**	1.58±0.18	1.61±0.20	1.44±0.23a c	1.27±0.21a c e	<0.001
**Right posterior cingulate cortex**	1.57±0.19	1.59±0.19	1.42±0.23a c	1.25±0.23a c e	<0.001

All values are indicated as mean ± standard deviation. p value indicates the value for the main effect of each group, as assessed with analyses of variance(ANOVA) for each variable. Statistics for post hoc 2-by-2 group comparisons are provided as significant differences:

a from CN at p<0.01;

b from CN at p<0.05.

c from EMCI at p<0.01;

d from EMCI at p<0.05;

e from LMCI at p<0.01.

From the ADNI-Go and ADNI-2 dataset, we selected all participants between 55–90 (inclusive) years of age who had completed, in the same visit, the following clinical, imaging and neuropsychological assessments: MRI, [^18^F]AV45 PET, [^18^F]FDG PET, Mini Mental State Examination (MMSE), Clinical Dementia Rating scale (CDR), Wechsler Memory Scale Logical Memory II, Alzheimer’s disease assessment scale (ADAS)-cog, Rey auditory verbal learning test (RAVLT), 30-item Boston Naming Test, Category Fluency (animal), and Trails Making Test (A & B), as well as the functional activities questionnaire (FAQ). Selected individuals were classified as CN, LMCI, EMCI and AD according to clinical and behavioral measures provided by ADNI at the time of the imaging study. Individual with Modified Hachinski Ischemia Score higher than four points were excluded during the screening phase. Furthermore, subjects with imaging evidence of clinically significant vascular changes were excluded from this analysis.

**Figure 3:**
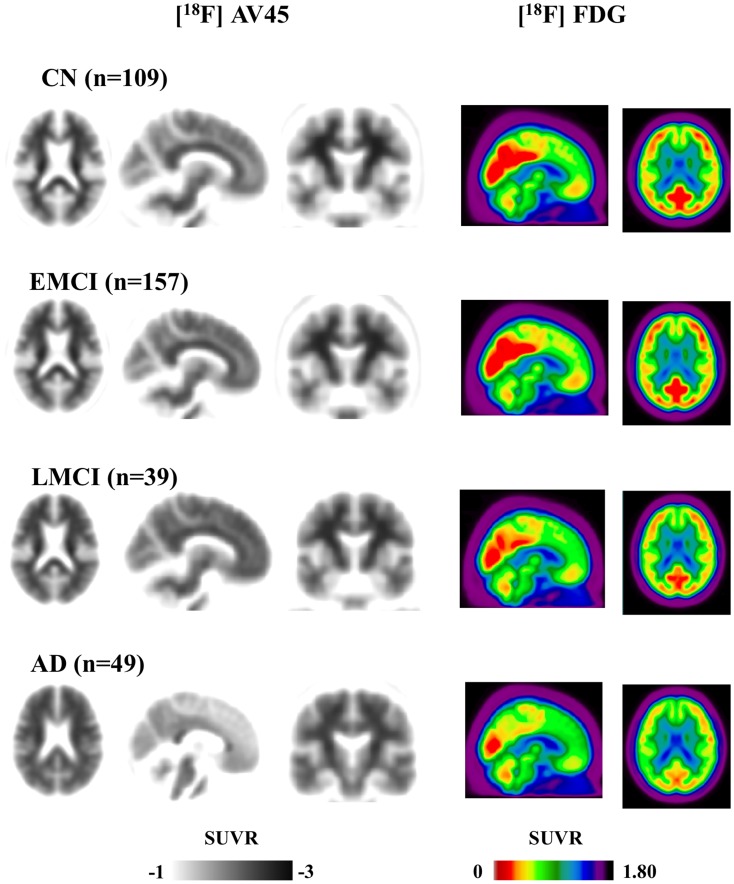
Comparison of global neocortical [^18^F]AV45 and [^18^F]FDG uptake among each group. Dissociation of amyloid deposition and hypometabolism in EMCI was shown by significant group difference in term of [^18^F]AV45 without group difference of [^18^F]FDG in EMCI versus CN. # means significant difference between groups (*p*<0.01), while NS represents no significant group difference(*p>*0.05).

**Figure 4:**
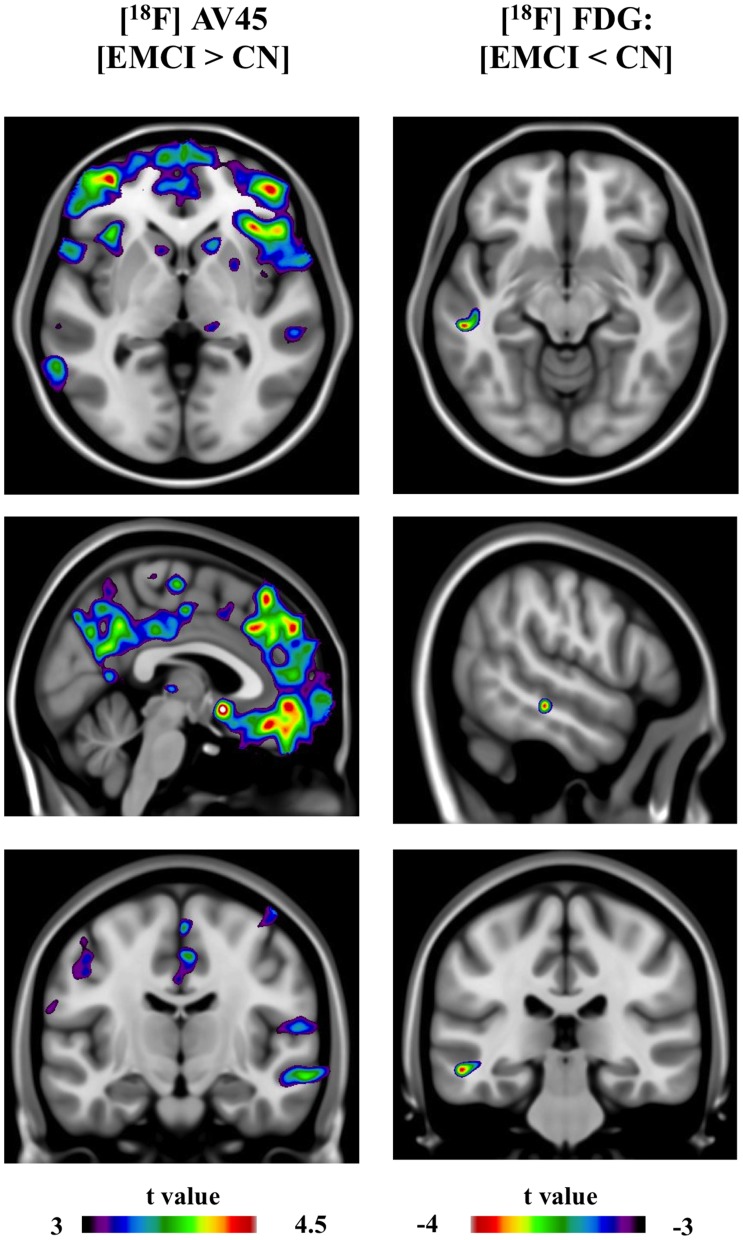
Images represent statistical parametric mapping depicting (spectrum color bars) t-statistical contrast overlaid on a group structural MRI. Right images represent voxel-based statistical differences comparing [^18^F]AV45 retention (EMCI > CN). Left images represents voxel-based statistical differences comparing [^18^F]FDG uptake (EMCI < CN).

### Classification Criteria

The criteria for CN included an MMSE score ranging between 24–30 (inclusive), and a CDR score of 0 (non-demented) [17], [18]. The criteria for EMCI included the presence of a subjective memory complaint, with an MMSE score between 24–30 (inclusive), objective memory loss as shown on scores on delayed recall of one paragraph from the Wechsler Memory Scale Logical Memory II (adjusted for age and education; ≥16 years: 9–11; 8–15 years: 5–9; 0–7 years: 3–6), a CDR of 0.5, preserved activities of daily living, and an absence of dementia [5], [19]. The criteria for LMCI subjects included the same criteria as EMCI, except for the greater objective memory loss measured by scores on delayed recall of Wechsler Memory Scale Logical Memory II (adjusted by age and education; ≥16 years: ≤8; 8–15 years: ≤4; 0–7 years: ≤2) [5]. In addition to the NINCDS/ADRDA criteria for probable AD, mild AD dementia subjects had MMSE scores between 20–26 (inclusive) and a CDR of 0.5 or 1.0 [20].

**Figure 5:**
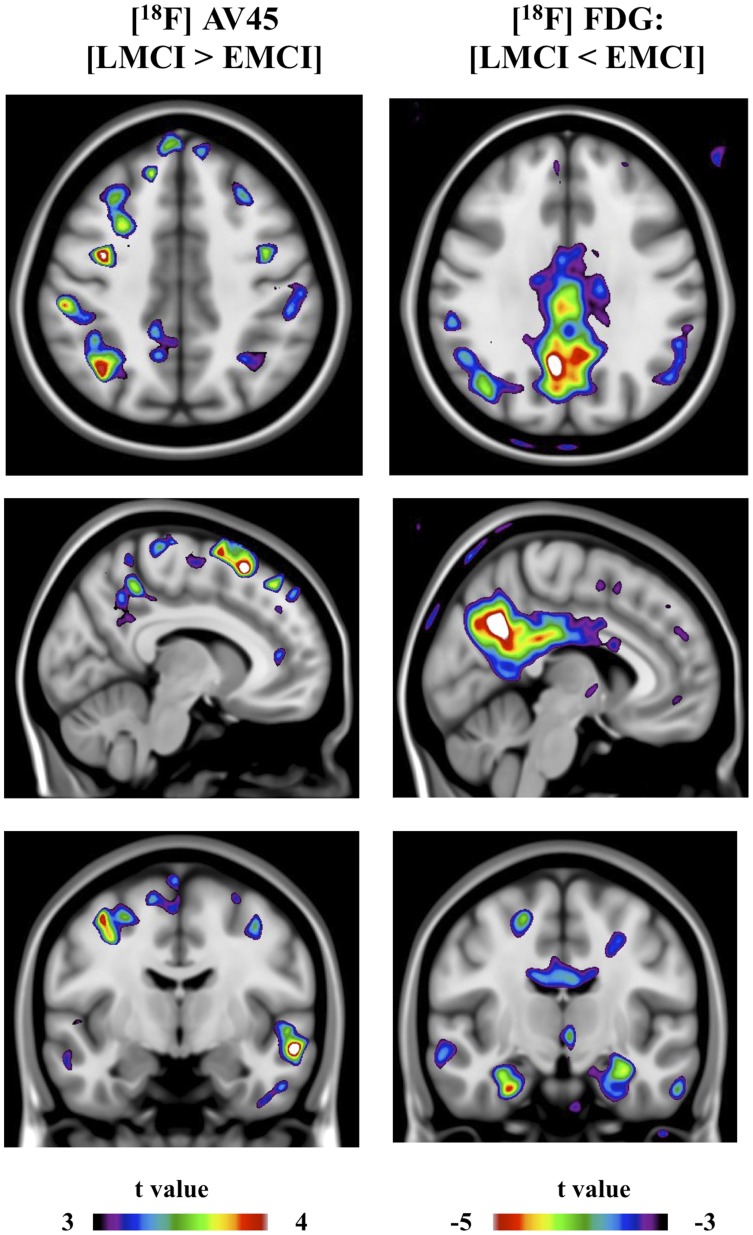
Images represent statistical parametric mapping depicting (spectrum color bars) t-statistical contrast overlaid on a group structural MRI. Right images represent voxel-based statistical differences comparing [^18^F]AV45 retention (LMCI > EMCI). Left images represents voxel-based statistical differences comparing [^18^F]FDG uptake (LMCI < EMCI).

**Figure 6:**
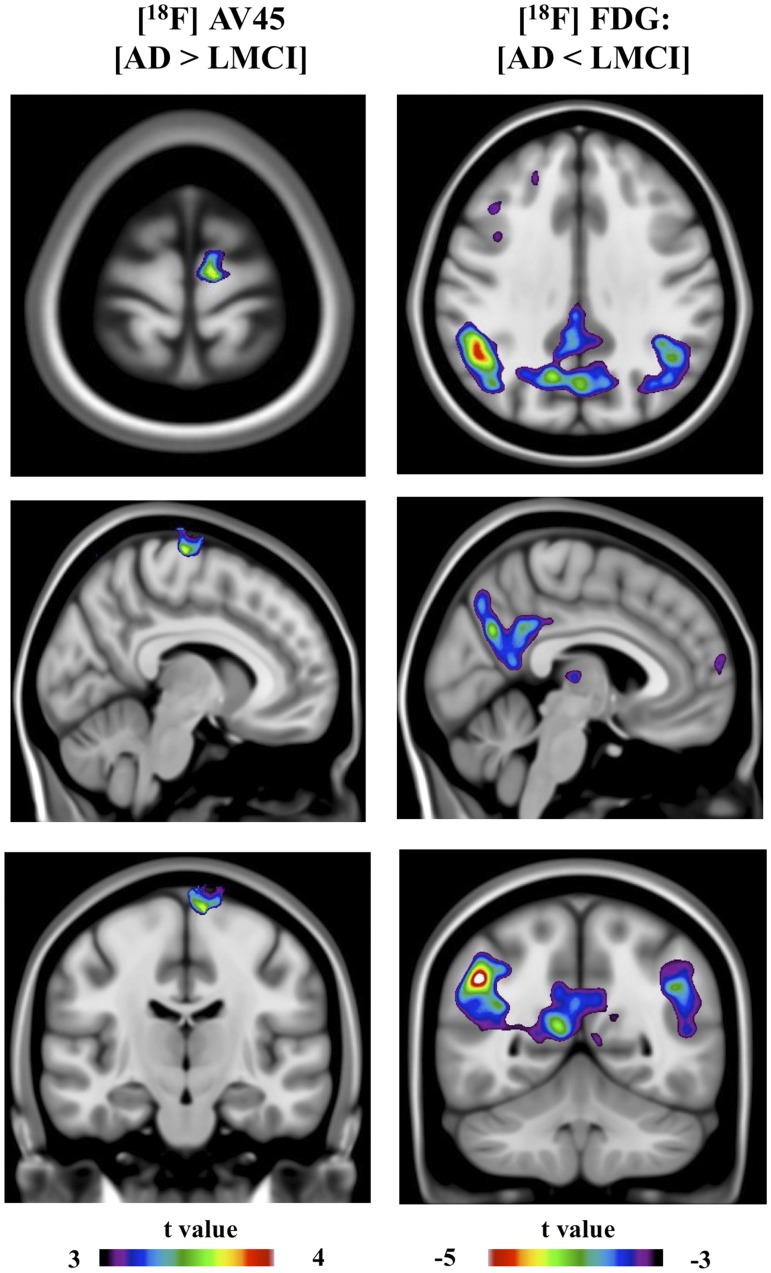
Images represent statistical parametric mapping depicting (spectrum color bars) t-statistical contrast overlaid on a group structural MRI. Right images represent voxel-based statistical differences comparing [^18^F]AV45 retention (AD > LMCI). Left images represents voxel-based statistical differences comparing [^18^F]FDG uptake (AD < LMCI).

### PET Methods

#### PET image acquisition

A detailed description of the [^18^F] AV45 and [^18^F]FDG PET image acquisition can be found at http://www.adni-info.org and http://www.loni.ucla.edu/ADNI/Data/ADNI_Data.shtml.

#### PET imaging processing

The image processing is summarized in Figure 1. In brief, T1 MRIs were corrected for non-uniformities and skull stripped. Subsequently, skull-stripped uncorrected MRIs were then linearly registered to the MNI space using mutual information and 9 parameters of transformation. Deformation fields (4 mm) were calculated for each individual scan. Next, the non-linearly registered MRIs were classified into grey matter (GM), white matter (WM) and CSF using the INSECT algorithm. Finally, ANIMAL algorithm extracted the cerebellum, hippocampi as well as the temporal, prefrontal, inferior parietal, anterior and posterior cingulate cortices.

#### Estimation of parametric images

Individual [^18^F]AV45 images were registered (see bellow) to the respective structural MRI using a 6 parameter mutual information algorithm. Individual brain regions were subsequently resampled to the PET native space. Uptake ratio (UR) images were calculated by dividing PET image by the median cerebellar grey matter count. Individual UR images were subsequently registered to the MNI space using the respective deformation fields. The images were then blurred using a 6 mm FWHM Gaussian kernel. [^18^F]FDG UR images were created in the same way as the [^18^F]AV45 except summed images were obtained using the pons as a reference region.

#### Estimation of global values

Global cortical [^18^F]AV45 retention was obtained from the median UR of the prefrontal, orbitofrontal, parietal, temporal, anterior cingulate and posterior cingulate/precuneus for each subject [21]. Global [^18^F]FDG uptake was formed by the median UR from the inferior parietal cortex, posterior cingulated gyrus, and temporal lobe including the hippocampus.

### Statistical Methods

Statistical tests were performed using SPSS 20.0 statistical software. Statistical significance was set at *p*≤0.05.

First, between-group comparisons of demographic and neuropsychological data were assessed using one-way ANOVA or chi-square (for gender) tests accordingly. Pairwise comparisons of CN, EMCI, LMCI and AD subjects were performed using an independence sample t-test.

Secondly, we interrogated [^18^F]AV45 and [^18^F]FDG UR at the voxel level in order to localize clusters of significant group difference. Voxel based group statistical differences were obtained by contrasting blurred [^18^F]FDG and [^18^F]AV45 UR images from all groups, after correcting for age, (2 at a time 6 total contrasts) using RMINC. RMINC is an imaging package that allows images files in the MINC to be analyzed with the powerful statistical environment R-statistic (http://www.r-project.org/). False discovery rate was used to threshold results for multiple comparisons [22]. Corrected threshold of significance was p<0.01 a t>3.5.

## Results

### Demographic and Neuropsychological Data

Three hundred fifty-four participants, including CN (n = 109), EMCI (n = 157), LMCI (n = 39) and AD (n = 49), were included in the present study. Demographic data and neuropsychological test scores are shown in Table 1. No significant differences in gender and education were noted among all groups. However, the age of EMCI is younger than CN and LMCI (*p*<0.001, t = 6.372, df = 263 for EMCI versus CN; *p* = 0.032, t = 2.159, df = 194 for EMCI versus LMCI).

When the four groups (CN, EMCI, LMCI and AD) were compared, significant differences were found for all neuropsychological scores (*p*<0.001). When compared to CN, EMCI individuals showed significant impairment on the majority of neuropsychological assessment measures, with the exception of trail making test A, recognition of AVLT and Boston naming test. The neuropsychological scores were significantly lower in EMCI patients compared to both LMCI and AD.

### Global Cortical [^18^F]AV45 Retention and [^18^F]FDG Uptake in Each Group

The global [^18^F]AV45 retention ratio values in each group are shown in Table 2 and Figure 2. EMCI patients showed significantly higher global [^18^F]AV45 retention compared to CN (1.40 versus 1.28, *p*<0.01) and lower amyloid retention compared to LMCI (1.40 versus 1.56, *p*<0.01). No significant differences were found between LMCI subjects and AD patients (1.56 versus 1.62, *p* = 0.39). Significance levels remained unchanged after applying age as a covariate.

The global and regional [^18^F]FDG uptake ratios in each group are shown in Table 2 and Figure 2. No significant differences in global [^18^F]FDG uptake ratio were found between EMCI subjects and CN individuals (1.42 versus 1.40, *p* = 0.233). In contrast, LMCI patients showed significantly lower global [^18^F]FDG uptake ratio compared to EMCI (1.28 versus 1.42, *p*<0.01) yet higher than AD (1.28 versus 1.14, *p*<0.01). Significance levels of group difference remained unchanged by the correction of age.

### Voxel-based Group Comparisons of [^18^F]AV45 Retention and [^18^F]FDG Uptake

[^18^F]AV45 and [^18^F]FDG average group images are represented in figure 3. SPM detected significantly higher [^18^F]AV45 retention in the EMCI subjects compared to CN individuals, mainly in the medial and ventral part of frontal lobe (e.g. medial prefrontal cortex, ventral lateral prefrontal cortex), anterior cingulate cortex (ACC), and posterior cingulate cortex (PCC)/precuneus (figure 4). Compared to EMCI, LMCI subjects showed significantly higher [^18^F]AV45 retention, most remarkably in dorsal lateral prefrontal cortex (DLPFC), and inferior parietal cortex (IPC), as well as middle temporal cortex (figure 5). Voxel-based analyses revealed a small but significant cluster with higher [^18^F]AV45 retention in right precentral gyrus in AD patients in contrast to LMCI individuals (figure 6).

SPM analyses revealed a small but significant cluster with lower [^18^F]FDG uptake in middle temporal gyrus in EMCI patients in contrast to CN (figure 4). Compared to EMCI, LMCI subjects showed significantly lower metabolism, most remarkably in bilateral posterior cingulate cortex (PCC)/precuneus, hippocampus and middle temporal gyrus, and, to a lesser extent, in the inferior parietal cortex (IPC) (figure 5). In contrast to LMCI, AD patients displayed significantly lower [^18^F]FDG uptake mainly in inferior parietal cortex (IPC) and posterior cingulate cortex (PCC)/precuneus, as well as in the temporal lobe (figure 6).

## Discussion

The present study explored, at a global and voxel-based level, brain cortical amyloid burden and metabolism using [^18^F]AV45 and [^18^F]FDG, respectively, within separate groups of CN, EMCI, LMCI and AD. We found that the global [^18^F]AV45 retention level in EMCI is intermediate between that of CN and LMCI, whereas no group differences in global amyloid retention were found between LMCI and AD. As for the global [^18^F]FDG uptake ratio, EMCI did not show significant differences when compared to CN, while hypometabolism was noticed in LMCI but not in EMCI. In terms of voxel based cortical [^18^F]AV45 retention, the main finding is that compared to CN, EMCI is associated with a significantly diffuse increased brain amyloid burden. In contrast, only one small hypometabolic cluster was found in the SPM contrast [EMCI < CN], though significant decrements in metabolism were noticed in PCC/precuneus and hippocampus in LMCI versus EMCI.

### Amyloid Deposition in EMCI

Abnormal amyloid load is detected in nearly a third of individuals older than 65 y.o. It is relatively well established that abnormal amyloid load is linked to APOE4 status as well as age [23]. A fraction of these individuals might carry, as recently described, reactive amyloidosis [24]. However, the interpretation of these finding remains elusive given the lack of long term longitudinal studies of cognitively normal individuals carriers of high load of amyloid pathology. The amyloid load of subjects with subjective cognitive impairment (SCI) was similar to that found in healthy elderly controls, remaining at a lower level compared with MCI and AD [7]. Longitudinal studies of non-demented older adults have shown that amyloid deposition increases slowly from cognitive normality and precedes cognitive impairment [25], [26]. Some of these reports have demonstrated an annual increase of amyloid deposition, in terms of [^11^C]PIB retention, of 0.9% per year in non-demented older adults, with the amyloid deposition localized to prefrontal, parietal, lateral temporal, and occipital cortices as well as anterior and posterior cingulate cortices [26]. However, it is not known how early in the disease course amyloid deposition can be detected. In the present study, the amyloid burden associated with EMCI is relatively lower than LMCI and higher compared to CN. This finding indicates that amyloid deposition characterizes EMCI stage and will possibly continue to accumulate during the progression from EMCI to LMCI [27].

A 2 year follow-up longitudinal [^11^C]PIB PET study showed amyloid load in mild AD remained relatively stable, suggesting that amyloid deposition in the brain might reach a plateau before the early clinical stages of AD [28]. The concept of amyloid plateau has been further supported by cross sectional studies showing similar amyloid load between MCI and AD, [7], [29], [30]. Interestingly, other longitudinal studies of MCI cohorts also suggest that the baseline amyloid load could be a predictor of dementia conversion [8], [9], [21], [31]. Together with the previous studies, the absence of group differences in terms of global amyloid retention between LMCI and AD in the present study indicates that amyloid load reaches a plateau at LMCI but not EMCI stage [9], [21], [29]. Therefore, the findings in the present study further support the dynamic biomarker model of AD, which posits that the amyloid plateau has already been reached in MCI [2].

Interestingly, we found higher amyloid load in ACC/PCC and the inferior part of the frontal lobe in EMCI versus CN, along with higher amyloid burden in temporal and parietal lobe, and superior part of frontal lobe in LMCI versus EMCI. This regional pattern of amyloid deposition is consistent with the pathological process described by autopsy studies where amyloid deposition progress from middle to lateral, from anterior to posterior and from basal to top of the brain [32].

### Brain Metabolism in EMCI

The lack of metabolism decrements in EMCI versus CN is possibly associated with a small magnitude in the [^18^F]FDG metabolism present in early MCI stages as well as the etiological variability within the MCI group. Indeed, variability across studies regarding brain metabolism in MCI occur due to the differences on the diagnostic criteria as well as the variations in clinical severity of the MCI subjects enrolled [12], [13], [33], [34]. Our estimates of global [^18^F]FDG excluded metabolically stable brain regions in AD in order to increase the sensitivity for detecting hypometabolism. In fact, [^18^F]FDG-PET studies have shown that medial temporal lobe (MTL), inferior parietal cortex and PCC are vulnerable to hypometabolism and thus appropriated for the identification of MCI, while the utility of other cortical deficits was deemed debatable [34]–[36]. Moreover, studies focusing on the temporal aspects of progression from MCI to AD conversion have suggested that impairment of [^18^F]FDG uptake in temporoparietal association cortices predicted a rapid progression to dementia in MCI patients and could, therefore, serve as a biomarker for the diagnosis of prodromal AD [10], [15], [37].

The most interesting finding of our study is a specific dissociation between brain amyloid deposition and metabolism in EMCI stage. The amyloid load in absence of hypometabolism further supports the view that amyloid deposition precedes neuronal dysfunction in the early stage of MCI [38]. Similarly, the dissociation between amyloid load and metabolic reductions was also demonstrated by another study, in which only 54% of the [^11^C]PIB positive MCI patients also showed [^18^F]FDG reductions [34].

Lower [^18^F]FDG uptake, primarily in bilateral PCC/precuneus and hippocampus, in LMCI versus EMCI supports that brain metabolic dysfunction develop in these regions during the MCI stage and thus might serve as a biomarker to monitor the disease progression from EMCI to LMCI. The present study also raises the question about the role of amyloid plaque formation in the synaptic dysfunction [39]. The group differences between EMCI and LMCI reported in this study support the assumption that brain amyloid deposition is linked with synaptic damage, especially in the PCC/precuneus and hippocampus during the MCI stage, which is of paramount importance vis à vis the use of [^18^F]FDG PET in clinical trials. Neuronal toxicity might be mediated by high oligomeric forms of Aβ_1–42_ [40]–[41].

### Implications for the Clinical Trial of Disease Modifying Prevention

It is accepted that the use of disease-modifying treatments during the dementia stage of AD may not be adequate, since extensive brain damage has already been established and that the best target population for disease-modifying therapy are those at the MCI stage [3], [42]. It is reasonable to assume that the maximal benefit of disease-modifying therapy targeting the amyloid pathophysiologic mechanisms underlying AD should be obtained in the earlier stage before the amyloid load reaches a plateau and irreversible pathological changes occur [3]. The findings in the present study therefore have several important implications. Firstly, the classification of EMCI is a useful concept in terms of early intervention with anti-amyloid therapy. In other words, anti-amyloid therapy should be administered in EMCI instead of LMCI in order to maximize the likelihood of slowing or halt amyloid deposition and thus the downstream pathophysiological processes including the decline of brain metabolism. It is likely that intervention in the late stage of disease would have to target other pathological processes since the amyloid load will have reached a plateau at LMCI stage [7]. Secondly, amyloid imaging can be utilized as a diagnostic biomarker for identifying EMCI individuals, whereas [^18^F]FDG PET imaging might be used as an endpoint biomarker to monitor the rate of disease progression and detect treatment effects in clinical trials for EMCI [3].

### Limitations of the Study

There are several limitations in the interpretation of the present study, which should be acknowledged. Firstly, since the ADNI-GO was initiated 2 years ago and [^18^F]AV45 and [^18^F]FDG imaging are to be performed only every two years from baseline, the present data are cross-sectional. Thus, the follow-up of these EMCI patients in ADNI-2 will be of paramount importance to record the real progress of amyloid deposition and metabolism. Secondly, although EMCI patients were carefully selected according to objective memory impairment and exclusion of any significant neurological disease other than suspected incipient AD, EMCI are still likely to encompass heterogeneous etiologies, thereby masking AD-specific findings to some degree. Since no genetic data of new subjects recruited from ADNI-GO and ADNI-2 are currently available for analysis, the impact of Apoe4 on the amyloid deposition is not included in the present study [43]. Finally, despite of the optimal binding to the fibrillary Aβ_1–42_ deposits, [^18^F]AV45 does not reveal hippocampal Aβ_1–42_ deposits.

### Conclusion

The present study explored the global and voxel-based cortical amyloid burden and metabolism within separate groups of CN, EMCI, LMCI, and AD. The present results indicate a dissociation between amyloid deposition and hypometabolism in EMCI. These results highlight the EMCI period as an optimal period for intervention with anti-amyloid therapies.
